# Solid State Photoreduction of Silver on Mesoporous Silica to Enhance Antifungal Activity

**DOI:** 10.3390/nano11092340

**Published:** 2021-09-09

**Authors:** Giulia Quaglia, Valeria Ambrogi, Donatella Pietrella, Morena Nocchetti, Loredana Latterini

**Affiliations:** 1 Nano4Light Lab, Dipartimento di Chimica, Biologia e Biotecnologie, Università degli Studi di Perugia, Via Elce di Sotto, 06123 Perugia, Italy; giulia.quaglia@studenti.unipg.it; 2 Dipartimento di Scienze Farmaceutiche, Università degli Studi di Perugia, Via del Liceo, 06123 Perugia, Italy; valeria.ambrogi@unipg.it (V.A.); morena.nocchetti@unipg.it (M.N.); 3 Dipartimento di Medicina, Università degli Studi di Perugia, Piazzale Gambuli, 06132 Perugia, Italy; donatella.pietrella@unipg.it

**Keywords:** silver-silica nanocomposite, photo-reduction process, solid state reaction, enhanced antimicrobial activity, improved material dispersibility

## Abstract

A solid-state Ultraviolet-photoreduction process of silver cations to produce Ag^0^ nanostructures on a mesoporous silica is presented as an innovative method for the preparation of efficient environmental anti-fouling agents. Mesoporous silica powder, contacted with AgNO_3_, is irradiated at 366 nm, where silica surface defects absorb. The detailed characterization of the materials enables us to document the silica assisted photo-reduction. The appearance of a Visible (Vis) band centered at 470 nm in the extinction spectra, due to the surface plasmon resonance of Ag^0^ nanostructures, and the morphology changes observed in transmission electron microscopy (TEM) images, associated with the increase of Ag/O ratio in energy dispersive X-ray (EDX) analysis, indicate the photo-induced formation of Ag^0^. The data demonstrate that the photo-induced reduction of silver cation occurs in the solid state and takes place through the activation of silica defects. The activation of the materials after UV-processing is then tested, evaluating their antimicrobial activity using an environmental filamentous fungus, *Aspergillus niger*. The treatment doubled inhibitory capacity in terms of minimal inhibitory concentration (MIC) and biofilm growth. The antimicrobial properties of silver–silica nanocomposites are investigated when dispersed in a commercial sealant; the nanocomposites show excellent dispersion in the silicon and improve its anti-fouling capacity.

## 1. Introduction

The search for an effective strategy to inhibit the growth of microorganisms on exposed surfaces is attracting increasing attention from the scientific community. In particular, materials and strategies to prevent the growth of environmental fungi, such as *Aspergillus niger*, are relevant due to respiratory diseases or complications caused by a fungi-contaminated environment [1,2]. In fact, excessive exposure to *Aspergillus niger* causes hypersensibility reactions in humans [3]. Thus, reducing or preventing surface contamination is an updated challenge since commercially available surface cleaning and disinfection procedures have modest effectiveness for medium- and long-term prevention [4].

Silver-based nanomaterials are among the most used additives to confer antimicrobial properties to materials for different applications, from biomedical devices and disinfectants [5] to food-packaging [6,7,8]. Many synthetic strategies have been proposed for the preparation of silver-based nanomaterials [9]. The chemical approach demands the use of reducing and/or stabilizing agents and the involvement of considerable amounts of solvents, which are, in many cases, special wastes to which appropriate treatments must be applied. Synthetic processes based on microorganisms, such as bacteria, fungi and algae, are currently being explored [10], but with these methods, nanomaterial samples with satisfactory polydispersity are obtained only after expensive purification steps. Physical preparation procedures, such as laser ablation, imply the necessity for highly controlled energy sources such as lasers [11].

The photo-induced reduction of silver cations in solution to obtain silver colloids was proposed a couple of decades ago [12,13]; data indicated that the process is assisted by electron donors present in the suspensions, which also act as reducing and protecting agents. Furthermore, the activation of the pre-formed silver nanoparticles or nanocrystals with Vis light is able to ensure a controlled growth of nanostructures with highly regular shapes, resulting in enhanced optical properties [14,15,16,17].

In most of the photo-induced processes, the radiation treatment has been carried out in suspension and the solvent plays a substantial role in assisting the diffusion of the reactive species. However, solvents must be disposed at the end of nucleation and/or growth processes. For example, Huang et al. produced silver nanoparticles via the photoreduction of silver nitrate in layered inorganic laponite clay aqueous suspensions [18].

Inorganic matrices, such as silicas [6,7,16,17,18,19,20] clays [18] and zeolites [16], have been used as templates to confine the growth of silver clusters formed by chemical reduction via hydrothermal method; in these materials, the inorganic matrices enable the tuning of their electronic properties [21]. In fact, it has been demonstrated that the size, shape and properties of silver nanostructures strongly depend on the pores/cavities present on the matrix as well as on the load of the metal precursor and the method to activate the precursor reduction and nucleation [17].

SYLOID AL-1FP is a synthetic mesoporous silica, which meets the specific test requirements of the United States Pharmacopeia-National Formulatory (USP-NF) for Silicon Dioxide, Japanese Pharmaceutical Excipients (JPE) for Hydrated Silicon Dioxide and the European Pharmacopeia (EP) for Colloidal Hydrated Silica; it presents a large superficial area (700 m^2^/g) with an internal and external porous structure resulting in an average pore volume of 0.4 mL/g and a mean diameter of 6–8 µm [22]. SYLOID AL-1FP can be used to improve the dispersibility of drugs or active substances. The silica matrix has been selected as a template for silver nanoparticle growth both for the presence of mesopores and for its large surface area, which allows a larger superficial exposure, increasing the possible interaction of silver with microorganisms [23].

Poly-dimethylsiloxanes, also known as silicones, are organic–inorganic polymers widely used as sealants and structural adhesives in construction and building applications [24]; in particular, acetoxy-silicone derivatives offer excellent adhesion to glass and ceramic surfaces and has therefore become an important sealant and adhesive in the glass and sanitary industries [25,26]. For these specific applications, silicones are exposed to high humidity, which might facilitate the growth of microbial colonies; despite commercially available acetoxy–silicones being formulated with antifouling and/or antimicrobial additives [27], the development of the black deposit growth, mainly due to *Aspergillus niger* [2] on the silicone junctions, is still an issue. Therefore, the development of antifouling sealants, to be used in environments with high sanitization requirements such as hospitals and nursing homes, is fundamental to enhancing the quality of this product.

In the present work, solid-state photochemically grown silver nanomaterials on SYLOID AL-1FP materials are used to enhance the antimicrobial properties of commercial silicone. The obtained materials are fully characterized and their antifouling action is tested against *Aspergillus niger*.

## 2. Materials and Methods

### 2.1. Materials 

SYLOID AL-1FP (SiO_2_) was kindly provided by Grace GmbH (Worms, Germany). Deionized water was obtained by a reverse osmosis process with a MilliQ system (Millipore, Rome, Italy). Silver nitrate and ethanol (reagent grade) were purchased from Sigma Aldrich (Milan, Italy) and used without purification.

### 2.2. Preparation of SiO_2_-Ag Samples

Silver nanoparticles were synthesized using SYLOID AL-1FP (SiO_2_) as a template. In particular, silver nitrate (200 mg) was dissolved in ethanol (20 mL) and then 1 g of SiO_2_ was added; the suspension was sonicated for 15 min and stirred for 24 h to facilitate the adsorption of silver on the surface of the matrix and the penetration into the pores. The resulting white powder (SiO_2_-Ag) was recovered by filtration, and it was stored in the dark at 37 °C for 24 h to remove the residual solvent. All the procedures to prepare the samples were carried out in dark conditions to avoid the interference of room and sunlight.

SiO_2_-Ag powder samples (ca. 20 mg) were exposed to UV radiation for two hours, using a medium pressure mercury lamp equipped with a band pass filter to select the wavelength at 366 nm. The obtained irradiated samples (named SiO_2_-Ag-Irr) were recovered and characterized.

### 2.3. Preparation of Silicone-Composite Samples

Silicone nanocomposite samples were obtained by mixing a commercially available acetoxy–silicone with the powder samples under investigation (SiO_2_-Ag or SiO_2_-Ag-Irr); as a reference, a composite with AgNO_3_ powder silicone was also prepared. The nanocomposites were extruded in discs (0.5 cm diameter, 0.1 cm height) using a homemade scaffold and the samples were left for two days in dark conditions at room temperature to let the silicone dry. The quantity of nanocomposites mixed to the silicone was based on the results of the Minimum Inhibitory Concentration (MIC) test (see below), in order to ensure a comparison of the data. Thus, the silicone discs with concentrations of silica-silver powder equal to 0.13 and 0.06 mg/mg_silicone_ for SiO_2_-Ag and SiO_2_-Ag-Irr, respectively, were prepared. The amount of AgNO_3_ was 0.12 mg/mg_silicone_.

### 2.4. Characterization

The optical properties of the solid samples were investigated through spectrophotometric measurements; the UV-Vis spectra were recorded by a Varian (Cary 4000) spectrophotometric, equipped with a 150 nm integration sphere and with a barium sulphate tablet used as a reference. The recorded spectra were analyzed with the Kubelka–Munk equation in order to make the comparison possible.

ATR-IR spectra in the 4000–400 cm^−1^ frequency range were acquired with an ALPHA compact FT-IR BRUKER, equipped with a diamond crystal; each spectrum was the average of 30 scans with a 2 cm^−1^ resolution.

X-ray powder diffraction (XPRD) patterns were taken with a Philips X’PERT PRO MPD diffractometer (Philips S.p.A., Milan, Italy) operating at 40 kV and 40 mA, with a step size of 0.0170 2θ° and a step scan rate of 30 s, using Cu Kα radiation and an X’Celerator detector (Philips S.p.A., Milan, Italy).

The morphology of the samples was investigated with a Philips 208 transmission electron microscope (Philips S.p.A., Milan, Italy) and with a FEG LEO 1525 scanning electron microscope (Carl Zeiss NTS GmbH, Oberkochen, Germany). FE-SEM micrographs were collected after depositing the powder samples or the silicon discs on a stub and sputter coating with chromium for 10 s.

For the TEM images, a drop of ethanol dispersion was deposited on a copper grid precoated with a Formwar film and was then evaporated in air at room temperature.

Metal analyses were performed with Varian 700-ES series inductively coupled plasma-optical emission spectrometers (ICP-OES) (Agilent Technologies, Mulgrave Victoria, Australia). A weighed amount of composite samples (about 20 mg) was dissolved in concentrated HF and HNO_3_ and the solution was brought to a final volume of 100 mL with distilled water. The solution, properly diluted, was analyzed by ICP.

Ζeta (ζ) potential measurements of a water dispersion of silica matrix and Ag-doped materials were carried out by Nicomp 380 ZLS PSS Particle Sizing Systems.

The release of silver from discs loaded with AgNO_3_, SiO_2_-Ag and SiO_2_-Ag-Irr was performed by immersing discs in tubes with 1 mL of sterile distilled water at 25 °C which were left under continuous stirring (50 rpm). At defined time intervals (7, 14, 28, 60, days), the supernatant was collected and the silver concentration was measured by ICP-OES estimating the amount (ppm) of Ag released as a function of time.

### 2.5. Antifungal and Antibiofilm Tests

*Aspergillus niger* (ATCC 16404) was used in the antimicrobial and antibiofilm activity assays. To prepare the inoculum, *Aspergillus niger* was grown in SAB (Sabouraud Dextrose Agar) medium with chloramphenicol on petri dishes for 72 h at 37 °C. The conidiospores formed on the surface of the fungus were collected and transferred into a sterile centrifuge tube with 5 mL of physiological solution. After sedimentation (5 min), the pellet was suspended in RPMI-1640, counted by hemocytometer and diluted in RPMI-1640 and 3-(Morpholin-4-yl)propane-1-sulfonic acid (MOPS) to obtain a concentration of 5 × 10^−4^ CFU/mL. A sterile microplate was filled with the inoculum of *Aspergillus niger* and SiO_2_-Ag and SiO_2_-Ag-Irr dispersed in RPMI-1640 and MOPS. The first line of the plate was used to control of the growth of the fungus and was filled with 200 µL of inoculum. The second line of the plate was utilized to control medium sterility (200 µL). The other lines were filled with 100 µL of inoculum and 100 µL of samples (irradiated and non-irradiated) diluted in saline solution at 1:2 scalar dilutions. The microplate was incubated at 37 °C for 24 h. After incubation, the plate was observed to determine the minimum concentration of silver nanoparticles able to inhibit the growth of *Aspergillus niger*. The Minimum Inhibitory Concentration (MIC) was established as the highest chemical agent dilution capable of inhibiting the fungal growth. Each test was conducted in triplicate.

Biofilm formation in the presence and absence of silver nanoparticles was analyzed in a static model on a flat bottom 96-well microplate. Silver nanoparticles with a concentration of one half, one fifth and equal to the MIC were used for the analysis and 100 µL of a suspension of *Aspergillus niger* in SAB and 2% sucrose were included in the microplate. After 48 h of incubation at 37 °C, supernatants were removed by aspiration with a syringe. The microplate wells were washed with 0.2 mL of PBS (phosphate buffered saline, pH = 7.4). The obtained biofilm was stained with 50 µL of 0.4% of Crystal Violet. After 15 min, the excess staining was washed two times with 0.2 mL of distilled water and 100 µL of ethanol was added. After 30 min, the measurement of the biofilm mass was carried out. Absorbance spectra at 570 nm were registered to observe the antifungal activity of silver nanoparticles.

The kinetics of biofilm growth were also analyzed for AgNO_3_, SiO_2_-Ag non-irradiated and irradiated silicone discs. *A. niger* was put in contact with the silicone discs containing the powder for 60 days and at defined intervals (0, 7, 14, 28, 60, days), the biofilm growth was analyzed using the same procedure described above.

## 3. Results

### 3.1. Material Preparation

Silver nanostructures were prepared through a solid-state photoreduction process of the cations adsorbed on silica. SYLOID AL-1FP (SiO_2_), a commercial mesoporous silica matrix, was selected as a support because it has a large superficial area, resulting in quite a good adsorption capability. Following the procedures described in the experimental section, the SiO_2_ matrix was loaded with silver cations, using AgNO_3_ as a precursor. The resulting white powder (SiO_2_-Ag) was then recovered and stored in the dark for further investigations (see below).

SiO_2_-Ag powder was then exposed to the 366 nm radiation from a filtered medium pressure Hg-lamp. The sample was exposed for two hours (SiO_2_-Ag-Irr), receiving ca. 1.3 Jcm^−2^; then SiO_2_-Ag-Irr was recovered and tested. The silver content in the samples was determined through ICP-measurements (Table 1). The difference in silver loading between the two samples was very small and within the experimental uncertainty. As expected, the photochemical method does not affect the silver content of the materials.

### 3.2. Characterization of Materials 

**Figure 1:**
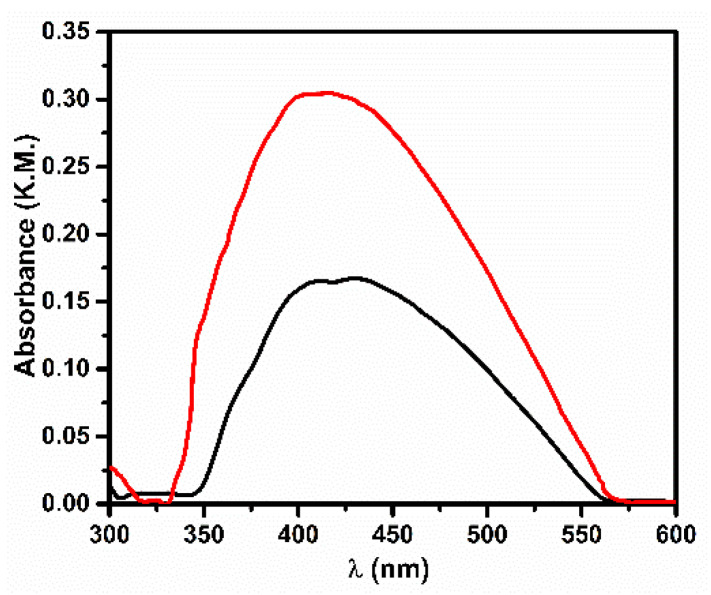
UV-Vis extinction spectra (Kubelka–Munk units) of SiO_2_-Ag before (black line) and after (red line) the 366 nm radiation.

The extinction spectrum of SiO_2_-Ag sample (Figure 1) shows a band centered at 430 nm, likely due to the plasmonic resonance (PR) of silver-based nanomaterials being formed as a consequence of the sonication treatment [28] during the precursor adsorption; the detailed computational analysis carried out by Allen et al. [29] suggests that multiple phases can be present. After exposure to 366 nm radiation, selectively absorbed by the silica matrix (see Figure S1a), an intensity increase of the PR band can be observed (Figure 1, red line). The blue-shift detected for the PR band suggests that UV treatment modifies the silver nanomaterials.

The morphological information on the samples was obtained through SEM and TEM imaging. Analysis of the SEM images (Figure S2) shows that the SiO_2_ matrix presents micrometric grains; on the surface of the matrix, small metal particles (in white) can be observed.

**Figure 2:**
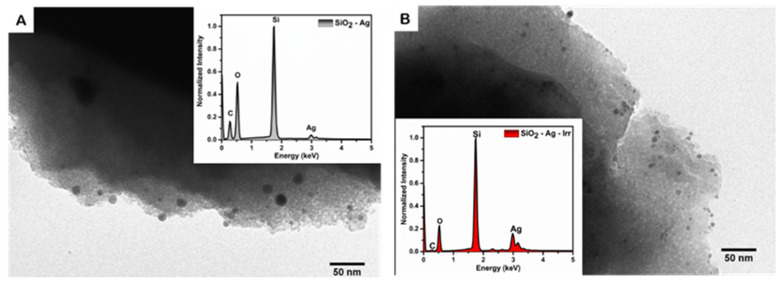
TEM images of SiO_2_-Ag (**A**) and SiO_2_-Ag-Irr (**B**). Insertion: EDX spectra of: (**A**) SiO_2_-Ag; (**B**) SiO_2_-Ag-Irr.

The analysis of TEM images enables us to gain information about the size and shape of the metal nanomaterials (Figure 2) before and after the irradiation. Both SiO_2_-Ag and SiO_2_-Ag-Irr samples show dark features on the edge of the micrometric matrix; these features are assigned to the silver nanostructures for the higher cross section with the electron beam. Before the exposure to the UV radiation, the sample SiO_2_-Ag presents metal-based particles with a diameter of 9–15 nm (Figure S3). After the radiation treatment, the number of nanoparticles per unit area has roughly doubled but their size is smaller (diameter about 5 nm), which is in agreement with the modifications observed in the PR spectrum.

EDX measurements revealed that, upon irradiation, the relative content of silver and oxygen are changed, resulting in an increased Ag/O ratio (Inset Figure 2). This observation supports the hypothesis that the mercury lamp radiation activated silica defects that were able to assist the metal reduction process. Thus, the change in Ag/O observed through the EDX measurements supports the occurrence of the photo-induced transformation of the Ag oxidation state.

The photo-induced modification of the silver-oxidation state is also confirmed by the zeta potential measurements. In agreement with the literature data [30], SiO_2_ presents a negative zeta potential value due to the presence of partially deprotonated silanol groups on the surface of the matrix in measurement conditions (water solution at pH = 6.5), which confer a negative charge distribution. As reported in Table 1, SiO_2_-Ag presents a positive zeta potential value, likely because of the adsorption of Ag ions on the silica surface; after irradiation, the surface of the material presents a negative charge; this observation indicates that silanol groups are back exposed to the water environments supporting the formation of Ag^0^ nanostructures.

Data indicate that the photoreduction process has occurred in the SiO_2_-Ag-Irr sample, thus providing a sustainable procedure to prepare metal nanoparticles. It is worth noting that the silica matrix plays an important role: in fact, the 366 nm radiation is mainly absorbed by SiO_2_; due to the presence of radiative and nonradiative defects in the nanostructured materials, which are ascribable to chemical deficiencies [31,32]. Moreover, it has been demonstrated that these local chemical defects can be activated by UV or even blue radiation, they are able to interact with chemical species adsorbed in close proximity [33] and they also generate reactive species [34,35].

ATR-IR spectral analysis shows interesting changes upon photo-induced reshaping treatment (Figure S4). The shifts of the peak at 1050 cm^−1^ (ascribable to the stretching Si-O-Si) indicate the interaction of silanol groups with silver metal (Si-O-Ag) [35]; furthermore, the shift of Si-OH stretching from 953 to 947 cm^−1^ in the irradiated sample can be assigned to the interactions between silica and the metal [36].

The XRD spectra of SiO_2_-Ag and SiO_2_-Ag-Irr (Figure S5) appear as a broad band with the equivalent Bragg angle at 2θ = 22°, which confirms that the silica support is amorphous. Moreover, no peaks attributable to silver could be detected, likely because of the low percentage of silver in the samples (below 1%) and the small size of the nanoparticles synthesized, which are under the sensitivity range of the instrument.

### 3.3. Antifungal Activity

**Figure 3:**
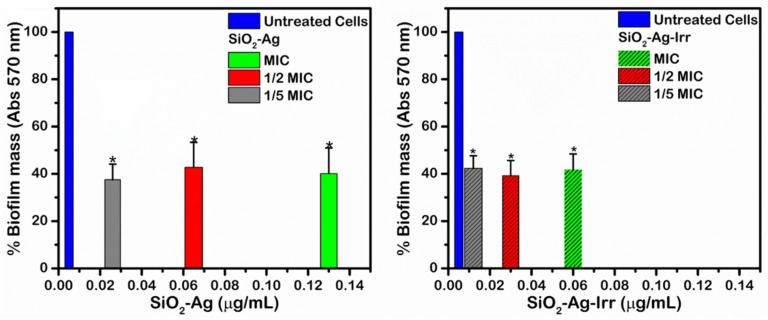
Biofilm mass growth of *A. niger* in presence of SiO_2_-Ag (**left**) and SiO_2_-Ag-Irr (**right**) compared to untreated cells (green striped column), at concentration equal to MIC (green columns), ½ MIC (red), and 1/5 MIC (grey).

To investigate the impact of photochemical treatments on the antimicrobial activity of silver nanocomposites, the Minimum Inhibitory Concentration (MIC, Table S1) determination was carried out using *Aspergillus niger* (Figure 3). These aerobic microorganism growths form a biofilm structure in oxygen-rich environment in the temperature range 15–40 °C that is classified as environmental mold [3]. The tests were conducted in vitro using the microdilution technique. A constant and known concentration of microorganism was suspended with scalar dilutions of the samples under investigation. The development of *Aspergillus niger* colonies in the different composite concentrations (see Table S1) enabled us to determine MIC values, which were 0.06 μg/mL for SiO_2_-Ag-irr and 0.13 μg/mL for SiO_2_-Ag. It is important to note that the antimicrobial activity of silver nanoparticles is improved after irradiation treatment. The data show that the SiO_2_-Ag-Irr sample has doubled inhibitory capacity compared to the non-irradiated one, since half the concentration of the SiO_2_-Ag-Irr sample is sufficient to prevent fungus growth. Since for both samples, the same silver loading was measured, the higher inhibitory efficiency of SiO_2_-Ag-Irr had to be related to the chemical and morphological changes that the radiation induced on silver.

*Aspergillus niger* is able to develop biofilm, mainly constituted by an extracellular matrix, where the colonies grow. The capacities of the materials to reduce biofilm development were also measured, using the composite concentration obtained from the MIC experiments (0.13 μg/mL for SiO_2_-Ag and 0.06 μg/mL for SiO_2_-Ag-irr, respectively). In Figure 3**,** the results of the assay are reported, which evidences that both SiO_2_-Ag and SiO_2_-Ag-Irr samples inhibit biofilm growth compared to the control experiment. It is worth highlighting that SiO_2_-Ag-Irr suppresses biofilm growth at lower concentrations compared to SiO_2_-Ag and that both composites control biofilm development even at concentrations lower than MIC values (*p* value lower than 0.05 calculated by *t*-test).

### 3.4. Preparation and Characterization of Silicone-Composites

In order to evaluate the applicability of the samples under investigation, SiO_2_-Ag and SiO_2_-Ag-Irr were dispersed in a commercially available acetoxy–silicone (Figure S6); for comparison, a silicone composite with AgNO_3_ was also prepared; however, AgNO_3_ does not homogenously disperse in silicone as indicated by visual inspections, which evidence the black grains. The dispersion of SiO_2_-Ag and SiO_2_-Ag-Irr in silicone does not change the physical properties, and the morphological characteristics of silicone are detected, as confirmed by the SEM images (Figure S7). The resulting composites are shaped in disc, as described in the experimental section and, then, the release of silver from the samples in distilled water is analyzed.

**Figure 4:**
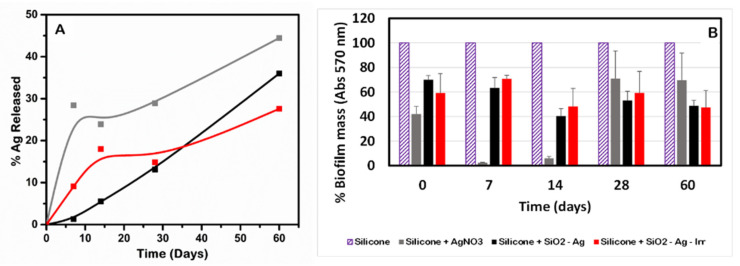
(**A**) Silver release from silicone functionalized with AgNO_3_ (grey line), SiO_2_-Ag (black line) and SiO_2_-Ag-Irr (red line) in function of time. (**B**) Biofilm mass growth of *A. niger* in presence of silicone discs (purple striped columns) and functionalized silicone with AgNO_3_ (grey columns), SiO_2_-Ag (black columns) and SiO_2_-Ag-irr (red columns).

The release of Ag ions from silicone discs loaded with SiO_2_-Ag and SiO_2_-Ag-Irr was monitored for 60 days. The concentration of Ag released was measured by ICP-OES. The data reported in Figure 4A highlight that silver ions are not completely released from the silicone even after 60 days. This observation confirms that the system is stable and capable of controlling the Ag release for a long time. As shown in Figure 4A, the silver release is slow and after 60 days reached 45%, 35% and 25% from silicone discs loaded with AgNO_3_, SiO_2_-Ag and SiO_2_-Ag-Irr, respectively. Interestingly, the release profile is different for all the samples.

Discs containing AgNO_3_ show an initial burst effect in inhibiting biofilm development, due to the prompt release of the Ag^+^ ions from the salt, largely water soluble, surface of the discs; indeed, the data display a reduced rate of Ag release after the 7th day. In the case of the composites containing silver nanoparticles, silver ions release occurs after metallic silver oxidation and only discs containing SiO_2_-Ag-Irr show a very small burst effect, maybe due to the larger surface exposure of these nanoparticles.

### 3.5. Biofilm Activity of Silicone-Composite Samples

The biofilm mass formed by *A. niger* in the presence of silicone-composite loaded with AgNO_3_, SiO_2_-Ag or SiO_2_-Ag Irr, monitored at different contact times, is also tested. The interesting result, shown in Figure 4B, is that the inhibition of the biofilm growth is also observed after 60 days of exposure for the composite samples. Moreover, comparing these results with those obtained for silicone discs containing AgNO_3_, it is possible to notice that discs containing AgNO_3_ prevent biofilm development for the first 14 days; however, at longer monitoring times, the data revealed the limited efficacy of discs containing AgNO_3_ to suppress biofilm growth, likely because most of the superficial Ag^+^ was released after 14 days. Thus, the presence of silver in nanoparticles greatly prolongs the antibiofilm action.

This result is probably due to the ability of the composites to release silver for prolonged times; while the corresponding bulk material (AgNO_3_) is available, its release is more rapid. In fact, as described in the previous paragraph, AgNO_3_ shows a high silver-release efficiency in the first 14 days, which is higher than that shown by the samples containing silver nanoparticles. Then, even if silver ions were still released, the antibiofilm activity of AgNO_3_ discs was no longer detected. Even if the silver release results are higher for discs containing SiO_2_-Ag, no differences in antibiofilm were observed between the samples containing silver nanoparticles. These observations can be explained with the complex antimicrobial mechanisms of silver nanoparticles, which are not only ascribed to silver ions release, but might also involve the formation of reactive oxygen species (ROS, such as hydroxyl radicals) due to the redox reaction of Ag^0^ [37] and to the adsorption of nanomaterials into the bacterial cell and the alteration of the bacterial membrane permeability [38].

## 4. Conclusions

In this work, silica–silver nanocomposites were synthetized by using a sustainable photochemical process. In particular, a commercially available mesoporous silica matrix (SYLOID AL-1FP) is used to support AgNO_3_; the exposure of the sample to 366 nm radiation (where the matrix mainly absorbs) for two hours resulted in a modification of the adsorbed metal. In particular, the plasmonic band of the metals shifted to shorter wavelengths becoming narrow and intense; IR spectra show the band shifted to a major wave number indicating changes in the silica–silver interaction. The superficial charge variation after the irradiation process confirms the reduction of silver ions; EDX spectra evidenced the increased content of silver with respect to oxygen, suggesting the occurrence of photoreduction processes; finally, TEM images show the presence of monodisperse silver nanoparticles in the matrix pores with a smaller size in the case of the irradiated sample. SiO_2_-Ag-Irr showed an enhanced antifungal activity compared to the non-treated samples, which is nicely correlated with the higher efficiency in silver ions release. The silica-silver nanocomposites are dispersed into silicone discs; the visual inspection and SEM imaging demonstrate that the nanocomposite materials are homogeneously dispersed in a commercial silicone without changing its aspect and surface morphology. The antibiofilm activity of silicone-composites is also tested; the data confirm that after 60 days there is an antimicrobial action against *A. niger*, while silicone loaded with AgNO_3_ shows an instantaneous antibiofilm activity that decreases after 14 days. The capability of silicone discs loaded with SiO_2_-Ag and SiO_2_-Ag-Irr to release silver ions is determined over 60 days. The data evidence the rather good ability of silica–silver nanocomposites to control silver release over time, thus limiting its uncontrolled dispersion, paired with an efficient antimicrobial power. The presented results highlight the exploitation of a sustainable, inexpensive, fast, treatment method, such as UV irradiation to activate the transformation of the material and to enhance its properties.

## Figures and Tables

**Table 1:** Silver content on the samples and Zeta-potential values.

Sample	Ag (ppm)	% Ag in the Sample	Zeta Potential (mV)
SiO_2_			−93.50
SiO_2_-Ag	2.98	0.62%	11.45
SiO_2_-Ag-Irr	3.09	0.66%	−69.10

## Data Availability

Data is contained within the article or supplementary material.

## Supplementary Materials

The following are available online at https://www.mdpi.com/article/10.3390/nano11092340/s1, Figure S1: UV-Vis reflectance spectrum of SYLOID AL1FP (A) and AgNO_3_ powder (B), Figure S2: SEM images of SiO_2_-Ag (A) and SiO_2_-Ag-Irr (B), Figure S3: Size distribution of SiO_2_-Ag (left) and SiO_2_-Ag-Irr (right), Figure S4: (A) ATR-IR spectra of SiO_2_ (blue line), SiO_2_-Ag (black line), SiO_2_-Ag-Irr (red line). (B) Normalized ATR-IR spectra at 436 cm^−1^, Figure S5: XRD spectra of SiO_2_-Ag (a) and SiO_2_-Ag-Irr (b), Figure S6: Photograph of functionalized silicone discs, Figure S7: SEM images of functionalized silicone section with AgNO_3_ (A); SiO_2_-Ag (B); SiO_2_-Ag-Irr (C), Table S1: Composite concentrations used for MIC determination.
