# Supplementary material for: Solid State Photoreduction of Silver on Mesoporous Silica to Enhance Antifungal Activity
Source: Nanomaterials (Basel). 2021 Sep 9;11(9):2340. doi: 10.3390/nano11092340 (PMC8465249; doi:10.3390/nano11092340)
Supplement: Supplementary file 1 [file nanomaterials-11-02340-s001.zip › nanomaterials-1365164-supplementary.pdf]

Supplementary Materials

Solid State Photoreduction of Silver on Mesoporous Silica to Enhance Antifungal Activity

Giulia Quaglia [1], Valeria Ambrogi [2], Donatella Pietrella [3], Morena Nocchetti [2] and Loredana Latterini [1,*]

[1] Nano4Light Lab - Dipartimento di Chimica, Biologia e Biotecnologie – Università degli Studi di Perugia, Via Elce di Sotto, 8, 06123, Perugia, Italy; giulia.quaglia@studenti.unipg.it (G.Q.)
[2] Dipartimento di Scienze Farmaceutiche, Università degli Studi di Perugia, Via del Liceo, 1, 06123, Perugia, Italy; valeria.ambrogi@unipg.it (V.A.); morena.nocchetti@unipg.it (M.N.)
[3] [c] Dipartimento di Medicina, Università degli Studi di Perugia, Piazzale Gambuli, 1 - 06132, Perugia, Italy; donatella.pietrella@unipg.it (D.P.)
* Correspondence: loredana.latterini@unipg.it; Tel.: +390755855583

Content:

- UV-Vis spectra of SYLOID AL1FP and $AgNO_3$. Page 1
- SEM images of solid powder samples. Page 1
- Size distribution histogram of silver nanoparticles on samples Page 1
- Zoom of the IR spectra of samples in the region of the Si-O stretching Page 2
- XRD spectra of samples Page 2
- Photographs of silicone discs loaded with $AgNO_3$ and samples Page 2
- SEM images of section of silicone discs Page 3

UV-Vis reflectance spectrum of SYLOID AL1FP

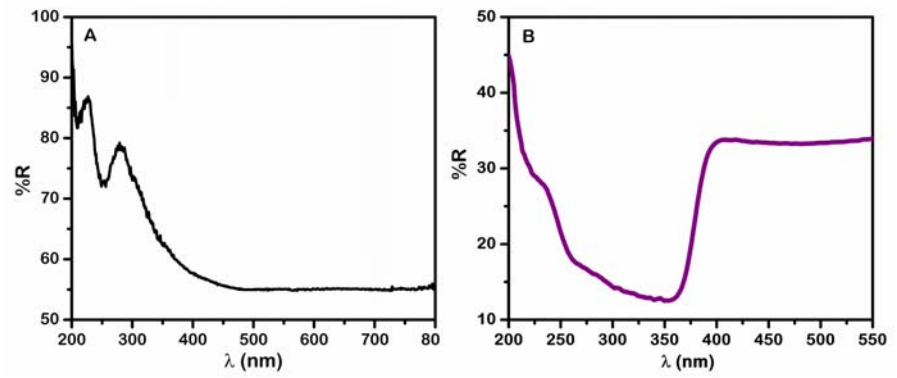

Figure S1: UV-Vis reflectance spectrum of SYLOID AL1FP (A) and $AgNO_3$ powder (B).

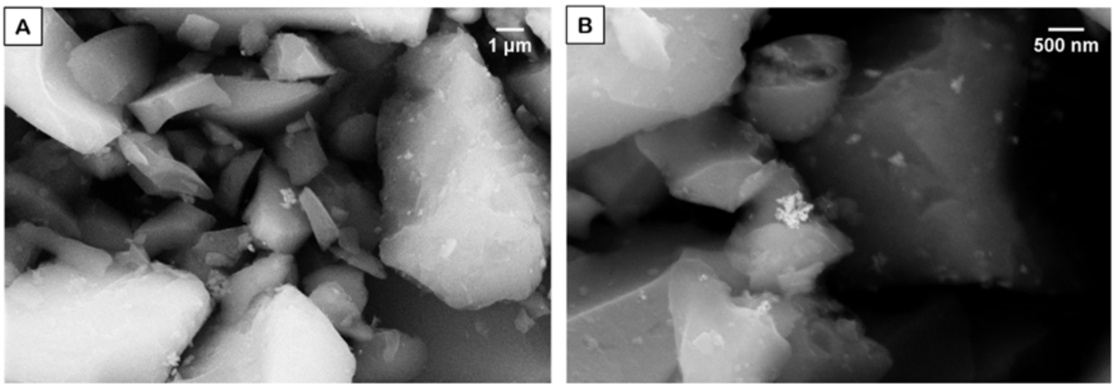

Figure S2: SEM images of SiO_2-Ag (A) and SiO_2-Ag-Irr (B).

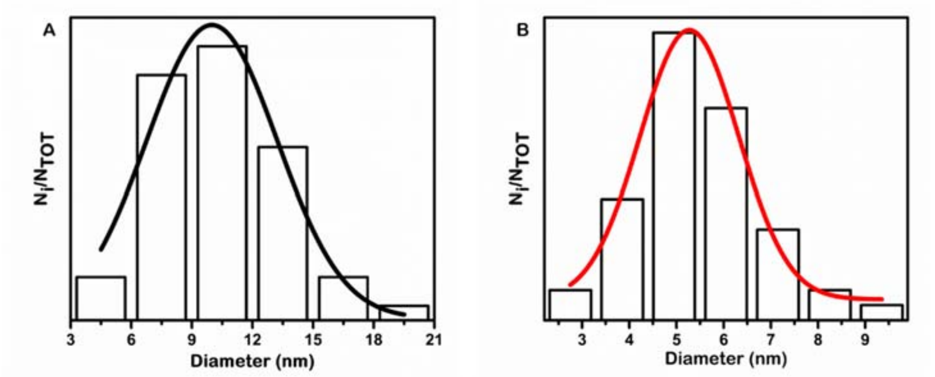

Figure S3: Size distribution of SiO_2-Ag (left) and SiO_2-Ag-Irr (right).

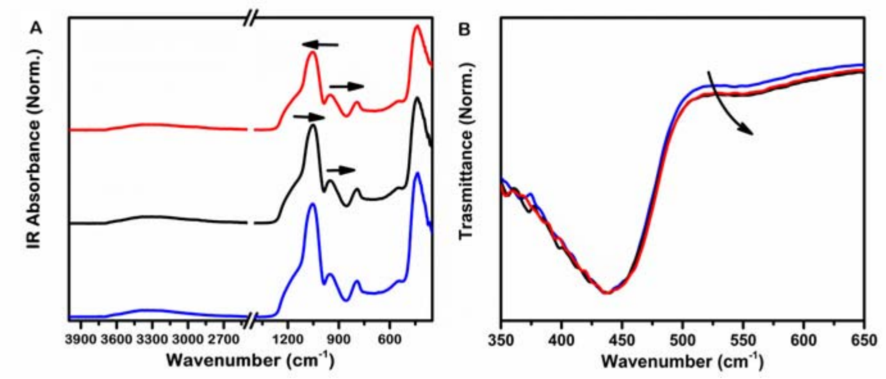

Figure S4: (A) ATR-IR spectra of SiO_2 (blue line), SiO_2-Ag (black line), SiO_2-Ag-Irr (red line). (B) Normalized ATR-IR spectra at 436 cm^{-1}.

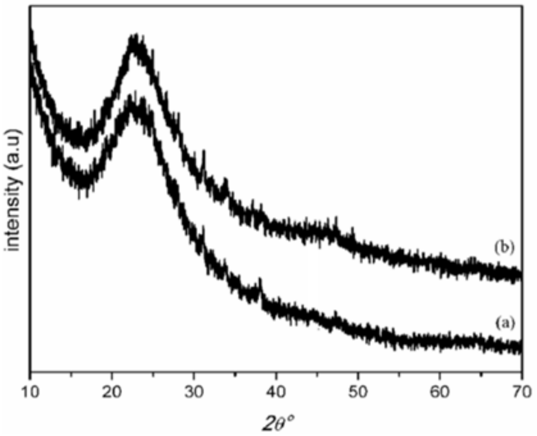

Figure S5: XRD spectra of SiO_2-Ag (a) and SiO_2-Ag-Irr (b).

Table S1: Composite concentrations used for MIC determination

	Tested composite concentration for MIC determination (µg/mL)										
Sample response	17.28	8.64	4.32	2.16	1.08	0.54	0.26	0.13	0.06	0.03	0.015	0.0075
SiO_2-Ag	X	X	X	X	X	X	X	V	V	V	V	V
SiO_2-Ag-Irr	X	X	X	X	X	X	X	X	V	V	V	V

Preparation and characterization of silicone-composites

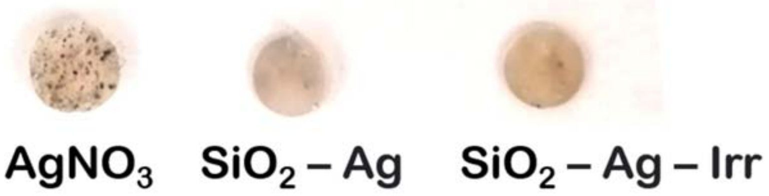

Figure S6: Photograph of functionalized silicone discs.

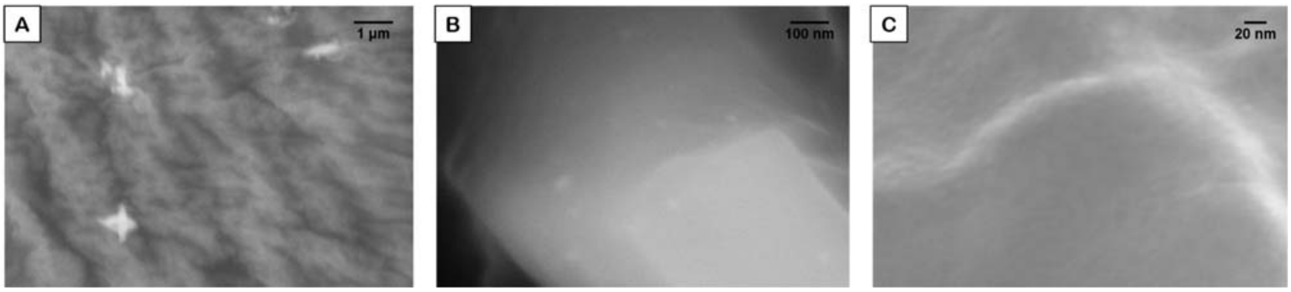

Figure S7: SEM images of functionalized silicone section with $AgNO_3$ (A); SiO_2-Ag (B); SiO_2-Ag-Irr (C).

Antifungal and antibiofilm tests

To prepare the inoculum, *Aspergillus niger* was grown in an SAB (Sabouraud Dextrose Agar) medium with chloramphenicol on petri dishes for 72 hours at 37°C. The conidiospores formed on the surface of the fungus were collected and transferred into a sterile centrifuge tube with 5 mL of physiological solution. After sedimentation (5 minutes), the pellet was suspended in RPMI-1640, counted by hemocytometer and diluted in RPMI-1640 and MOPS to obtain a concentration of 5×10^{-4} CFU/mL. A sterile microplate was filled with the inoculum of *Aspergillus niger* and SiO_2-Ag and SiO_2-Ag-Irr dispersed in RPMI-1640 and MOPS. The first line of the plate was used to control the growth of the fungus and filled it with 200 µL of inoculum. The second line of the plate was utilized to control medium sterility (200 µL). The other lines were filled with 100 µL of inoculum and 100 µL of samples (irradiated and non-irradiated) diluted in saline solution at 1:2 scalar dilutions. The microplate was incubated at 37°C for 24 hours. After incubation, the plate was observed to determine the minimum concentration of silver nanoparticles able to inhibit the growth of *Aspergillus niger*. The Minimum Inhibitory Concentration (MIC) is established as the highest chemical agent dilution capable of inhibiting the fungal growth. Each test was conducted in triplicate.

Silver nanoparticles with a concentration of one half, one fifth and equal to the MIC were used for the analysis and 100 µL of a suspension of *Aspergillus niger* in SAB and 2% sucrose were included in the microplate. After 48 hours of incubation at 37°C, supernatants were removed by aspiration with syringe. The microplate wells were washed with 0.2 mL of PBS (phosphate buffered saline, pH=7.4). The biofilm obtained was stained with 50 µL of 0.4% of Crystal Violet. After 15 minutes, the excess staining was washed two times with 0.2 mL of distilled water and 100 µL of ethanol was added. After 30 minutes, the measurement of biofilm mass was carried out. Absorbance spectra at 570 nm were registered to observe the antifungal activity of the silver nanoparticles.

The kinetics of biofilm growth were also analyzed for silicone functionalized with $AgNO_3$, SiO_2-Ag and SiO_2-Irr. *A. niger* was put in contact with the silicone discs containing the powder for 60 days

and, at defined time intervals (0, 7, 14, 28, 60, days), the biofilm growth was analyzed with the same procedure described above.
